# Simulation-based optimization and experimental comparison of intracranial T2-weighted DANTE-SPACE vessel wall imaging at 3T and 7T

**DOI:** 10.1002/mrm.30203

**Published:** 2024-07-06

**Authors:** Matthijs H.S. de Buck, Aaron T. Hess, Peter Jezzard

**Affiliations:** 1 Wellcome Centre for Integrative Neuroimaging, FMRIB Division, Nuffield Department of Clinical Neurosciences, University of Oxford, Oxford, UK; 2 Spinoza Centre for Neuroimaging, Netherlands Institute for Neuroscience, Royal Netherlands Academy for Arts and Sciences (KNAW), Amsterdam, The Netherlands

**Keywords:** black-blood, DANTE-SPACE, 7 Tesla, Vessel wall imaging

## Abstract

**Purpose:**

T2-weighted DANTE-SPACE (Delay Alternating with Nutation for Tailored Excitation — Sampling Perfection with Application optimized Contrasts using different flip angle Evolution) sequences facilitate non-invasive intracranial vessel wall imaging at 7T through simultaneous suppression of blood and CSF. However, the achieved vessel wall delineation depends closely on the selected sequence parameters, and little information is available about the performance of the sequence using more widely available 3T MRI. Therefore, in this paper a comprehensive DANTE-SPACE simulation framework is used for the optimization and quantitative comparison of T2-weighted DANTE-SPACE at both 7T and 3T.

**Methods:**

Simulations are used to propose optimized sequence parameters at both 3T and 7T. At 7T, an additional protocol which uses a parallel transmission (pTx) shim during the DANTE preparation for improved suppression of inflowing blood is also proposed. Data at both field strengths using optimized and literature protocols are acquired and quantitatively compared in six healthy volunteers.

**Results:**

At 7T, more vessel wall signal can be retained while still achieving sufficient CSF suppression by using fewer DANTE pulses than described in previous implementations. The use of a pTx shim during DANTE at 7T provides a modest further improvement to the inner vessel wall delineation. At 3T, aggressive DANTE preparation is required to achieve CSF suppression, resulting in reduced vessel wall signal. As a result, the achievable vessel wall definition at 3T is around half that of 7T.

**Conclusion:**

Simulation-based optimization of DANTE parameters facilitates improved T2-weighted DANTE-SPACE contrasts at 7T. The improved vessel definition of T2-weighted DANTE-SPACE at 7T makes DANTE preparation more suitable for T2-weighted VWI at 7T than at 3T.

## 1 Introduction

Large-artery intracranial occlusive disease is a common, and possibly the most prevalent, cause of stroke globally.^1^ This mainly relates to various arteries in and around the Circle of Willis (CoW), with the distal internal carotid arteries (ICAs), basilar artery (BA), and middle cerebral arteries (MCAs) being the most involved.^2,3^ Intracranial MR vessel wall imaging (VWI) aims to non-invasively characterize the location, size, and composition of intracranial pathology such as plaque burden in arterial vessel walls (VWs). For this, MR VWI aims to delineate the inner and outer boundary of the VW through suppression of signals both within and around the VWs,^4^ while maintaining high VW signal.^5^ Additionally, MRI-based VWI can distinguish different plaque components through variable signal contrasts relative to healthy VW tissue. Multi-contrast VWI, such as via separate T1- and T2-weighted acquisitions, is required for optimal differentiation of various vasculopathies.^6^

The DANTE-SPACE sequence has previously been introduced for T2-weighted VWI at 7T.^7^ It achieves intracranial VW delineation using DANTE (delay alternating with nutation for tailored excitation) preparation for suppression of blood and CSF, followed by a variable flip angle turbo-spin-echo (SPACE)^8^ readout module. Viessmann et al.^7^ optimized the SPACE flip angle train for sharp VW depiction at 7T and used Bloch equation simulations to propose an initial DANTE implementation. However, they also reported substantial inter- and intrasubject variations in the achieved contrasts, as well as a 50% reduction in the wall-to-lumen contrast-to-noise ratio (CNR) compared to regular SPACE. These limitations to the contrast levels and their homogeneity could hinder the clinical implementation of this T2-weighted VWI sequence, which requires consistently high signal levels for clear delineation and visualization of VW architecture and pathology. Furthermore, the limited achieved contrasts restrict further improvement to the resolution and scan time of the sequence.

At 7T, the T2-weighted DANTE-SPACE sequence benefits from the increased SNR and longer T1 relaxation times associated with higher magnetic fields to achieve vessel delineation relative to both the internal blood and the surrounding CSF. However, 7T MRI is currently not a part of routine clinical practice due to the limited number of available 7T scanners, higher associated costs, additional safety considerations, and remaining limitations from regulatory bodies.^9–11^ Conversely, 3T MRI, which is considered the minimum magnetic field strength for intracranial VWI,^5^ is widely available in a clinical setting.

At 3T, DANTE-SPACE has more frequently been used as a 3D T1-weighted intracranial VWI sequence,^12–19^ where compared to T2-weighted acquisitions it benefits from relatively short acquisition times and increased DANTE efficiency due to a shorter TR, and lower native CSF signal. In 2021, Zhang et al.^20^ acquired 3D T2-weighted DANTE-SPACE images at 3T as part of a comparison of different CSF-suppression methods for T2-weighted SPACE. Using the same DANTE parameters as previously used for T1-weighted DANTE-SPACE at 3T^13,14,16^ (consisting of 150 DANTE pulses of 8°), they reported insufficient CSF suppression for accurate VW delineation.^20^ In a different study, Wang et al.^4^ used DANTE preparation in 2D T2-weighted turbo-spin-echo acquisitions at 3T and reported overall satisfactory CSF suppression around the major intracranial arteries, resulting in a 28% increase in CSF-to-wall contrast for proton-density-weighted DANTE-SPACE. However, no conclusive information is available about the optimization and performance of 3D intracranial T2-weighted DANTE-SPACE at 3T.

Further, no information is currently available about the relative performance of T2-weighted DANTE-SPACE at 3T versus 7T. In addition to various advantages, higher field strengths also introduce limitations. Increasing SAR leads to more restrictive limits on the allowed sequence parameters, while smaller transmit field coverage limits the suppression of inflowing blood.^21^ Finally, while the typically longer T1 and shorter T2 times at 7T benefit CSF suppression, they also result in reduced VW signal.^22^ Therefore, it is not obvious whether and by how much T2-weighted DANTE-SPACE benefits from higher field strengths.

In this work, therefore, optimized T2-weighted DANTE-SPACE implementations at both 3T and 7T are proposed and quantitatively compared. For this, we used a recently developed simulation framework for the DANTE-SPACE sequence,^22^ which includes physiological processes such as pulsatile flow velocity variation, varying flow directions, intravoxel dephasing, diffusion, and RF field (B1^+^) effects.

Previous literature has also used simulations for DANTE optimization,^4,7,12,16,20,23,24^ but has excluded the effects of physiological processes such as CSF and VW pulsation and variations in the B1^+^ field. Therefore, this recent simulation framework facilitates a more comprehensive optimization of the T2-weighted DANTE-SPACE sequence for improved vessel delineation. Furthermore, including measures of B1^+^ inhomogeneity in the simulations makes it possible to assess and optimize the benefits of implementing parallel transmit (pTx) RF shims during DANTE for improved B1^+^ coverage in the vessels in the neck^25^ for improved suppression of signal from inflowing blood.

This paper first uses the recent DANTE-SPACE simulation framework to propose optimized T2-weighted DANTE-SPACE protocols for three scenarios: at 3T; at 7T without pTx; and at 7T with pTx. Subsequently, *in vivo* data from healthy volunteers at both field strengths are used to quantitatively compare the performance of T2-weighted DANTE-SPACE in all three scenarios.

## 2 Methods

### 2.1 Simulations

Using the previously presented extended phase graph^26^ DANTE-SPACE simulation framework,^22^ predicted signal levels and contrasts were simulated for VW, CSF, and blood, based on the tissue properties shown in Table 1. The physiological parameters and relaxation parameters at 7T are based on various original sources^7,27–32^ as previously described.^22^

T1 and T2 relaxation times at 3T were estimated based on various sources. VW relaxation times were based on carotid artery measurements^27^; literature values^33,34^ were used for blood at 3T; for CSF at 3T, the same T1 as at 7T was used based on previous studies^29,35^ that found it to be constant for different field strengths, while the CSF T2 at 3T was interpolated from measurements taken at various other field strengths.^30,35–37^

B1^+^ variations in different parts of the vasculature at 7T were included based on a previously presented database of 7T multi-channel pTx B1^+^ flip angle maps in both the head and the neck.^25^ Because of the improved transmit field homogeneity and extent at 3T compared to 7T,^38^ B1^+^ fields were assumed to be homogeneous in all 3T simulations. Temporal velocity variations due to pulsatile CSF were based on literature-sourced time-varying CSF flow measurements at the third ventricle (with an average flow velocity of 0.37 cm/s^31^). Inflowing blood was modeled on the cardiac pulsatility of blood in the internal carotid arteries.^32^ CSF and VW signal levels were calculated based on the values during the second TR to account for magnetization history effects from previous repetitions.^22^

Using this simulation set-up, parameter sweeps were used to determine the DANTE parameters that optimize the VW-CSF and VW-blood contrasts.

### 2.2 Data acquisition

DANTE-SPACE data were acquired at both 3T and 7T for the same six healthy volunteers (24–57 y old; 33 y average; five male) under an agreed institutional ethics agreement. 3T data were acquired using a Siemens (Erlangen, Germany) Prisma scanner with a 32-channel receive head coil. 7T data were acquired on a Siemens Magnetom scanner using a Nova Medical (Wilmington, MA) 8Tx/32Rx head coil. The order in which different protocols were acquired was varied between subjects to ensure unbiased comparisons. Manual (non-oblique) slice positioning was used for each subject independently at 3T and 7T.

Basic scan parameters were based on the T2-weighted 7T protocol used by Viessmann et al.^7^ This includes data acquired at a resolution of 0.47 × 0.47 × 1.0 mm (reconstructed to 0.23 × 0.23 × 1.0 mm for improved visualization) with a total FOV of 240 × 180 × 176 mm. Other SPACE parameters at 7T include TR/TE_eq_ = 2620/165 ms, echo spacing = 4.62 ms, echo train length = 74 pulses (342 ms), GRAPPA *R* = 4 in the left–right phase-encode direction (24 × 24 calibration region), band-width = 465 Hz/pixel, and a total scan time of 11:32. At 3T, simulations were first used to propose a T2-weighted SPACE protocol. This consisted of optimizing the TR and the SPACE echo spacing, after which the echo train length was adjusted to match the total scan time at 7T. After this SPACE optimization, additional simulations were used for a separate DANTE parameter optimization (while adopting the 3T-optimized SPACE protocol).

At 7T, a FOV in the head-foot direction of 176 mm could be used with non-selective SPACE refocusing pulses without resulting in wrap-around artifacts due to the inherently limited spatial extent of the transmit and receive fields at ultra-high field.^38,39^ To suppress longitudinal wrap-around artifacts at 3T due to the larger spatial extent of the B1 fields into the neck, an additional spatial saturation pulse was used between the DANTE-modules and the SPACE-readouts. Preliminary in vivo DANTE-SPACE data using various FOV sizes in the longitudinal direction were used to assess the required size and location of this spatial saturation region, as shown in Figures S1 and S2. Based on this, a spatial saturation pulse covering a 70 mm slab below the FOV was included between the DANTE preparation module and the SPACE readout for signal suppression inferior to the 176 mm FOV used for all acquisitions at 3T.

To account for the lower spatial variation in the receive coil sensitivity profiles at 3T compared to 7T, a lower GRAPPA acceleration factor was used (*R* = 3). Most other SPACE parameters, including the total scan time, FOV, matrix size, equivalent TE, and receive bandwidth were matched between 3T and 7T (see Section 3 below for a full overview of the parameters). The SPACE echo spacing (defined as the time between consecutive pulses) and the overall TR at 3T were selected based on simulation results. After all SPACE parameters were selected, the echo train length was adjusted to achieve the same total scan time at 3T as at 7T.

### 2.3 Reconstruction

All acquisitions were reconstructed using the same offline pipeline in MATLAB (Mathworks, Natick, MA) to facilitate reconstruction and direct comparison in SNR units.^40^

First, 2D-multislice GRAPPA^41^ reconstruction was applied using a 3 × 2 kernel. Minor spiking artifacts were encountered in the highest spatial frequencies along one side of the left–right k-space direction of the acquisitions at 7T (due to an issue with a loose gradient cable). To suppress these artifacts, the outer 9% of measured k-space containing the observed spikes was replaced using a projection onto convex sets (POCS)^42,43^ partial Fourier correction method.

The reconstructed k-space was zero-padded to obtain the desired reconstructed resolution of 0.23 × 0.23 mm in-plane. A 2D Fermi filter (using an exponent denominator of 8) was applied to suppress ringing artifacts from zero-padding.

Additional noise-only measurements were acquired for every acquisition and used for reconstruction in B1-weighted SNR units,^40,44^ calculated as

(1) $${\text{SNR}}_{\text{B}1\text{w}}=\sqrt{2/R}\frac{\left|S^{H}N^{-1}I_{\text{ch}}\right|}{\sqrt{S^{H}N^{-1}S}},$$

where SNR_B1w_ denotes the B1-weighted reconstructed image with pixel intensities in SNR units, calculated from the ESPIRiT^45^ coil sensitivities *S*, the bandwidth-scaled noise correlation matrix *N*, the multi-coil image data *I*_ch_, and the undersampling factor *R*. Superscript *H* denotes the conjugate transpose operation. ESPIRiT receive coil sensitivities were estimated using the Berkeley Advanced Reconstruction Toolbox (BART; v0.4.02).^46,47^

Datasets acquired from each subject at 3T and 7T were rigidly registered using three-dimensional translation and in-plane rotation (yaw). The other two rotation orientations (pitch and roll) were excluded from the registration to prevent confounds in the calculated image properties due to interpolation-induced blurring when rotating along anisotropic voxel directions. Although this method of image registration means that full slices may not be properly registered across field strengths, it should ensure that local patches of the slice can be compared with accuracy. This is all that is necessary to compare local VWs across the two field strengths, despite slight possible differences in the original slice locations and orientations.

### 2.4 Quantification of vessel visibility

**Figure 1:**
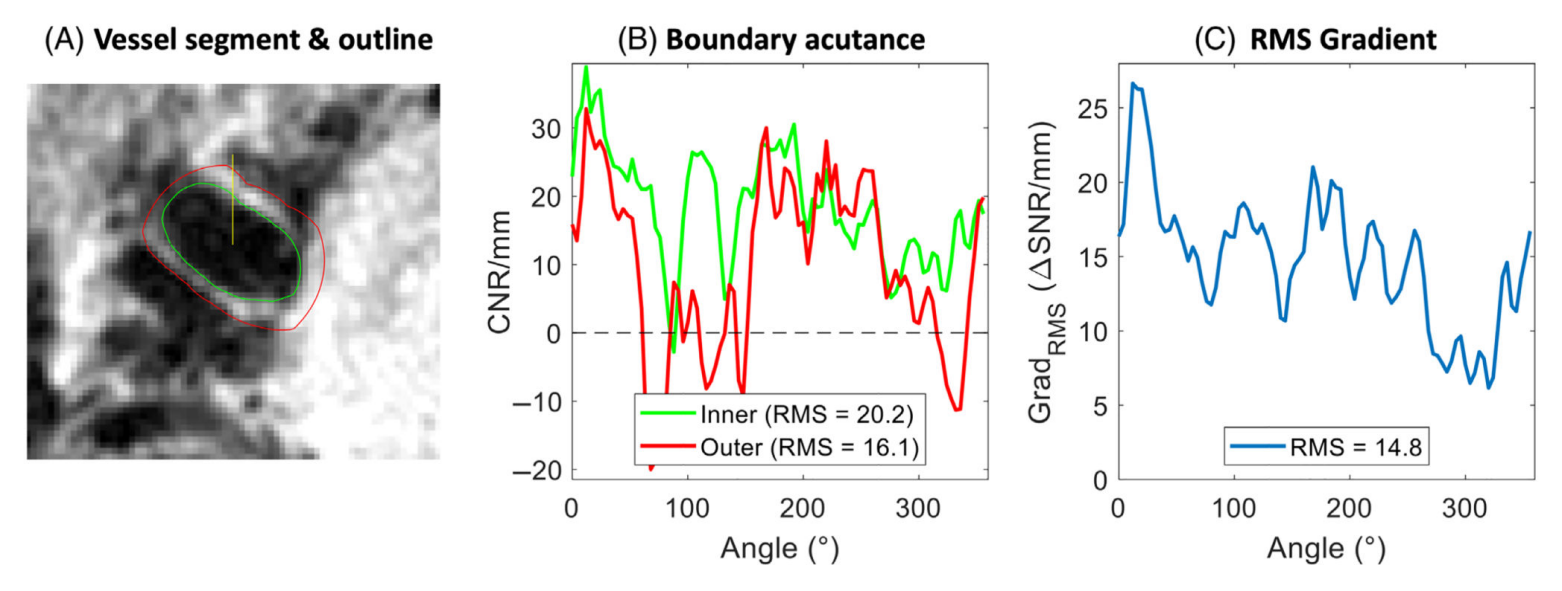
Example vessel delineation and acutance calculation. (A) Axial vessel segment and the estimated inner (green) and outer (red) vessel boundaries. (B) Calculation of the inner and outer boundary acutance at each of the 90 radial directions, sampled clockwise starting at the vertical yellow line in (A). RMS values of the two contrasts are included in the legend. (C) Corresponding RMS gradient values of the vessel wall along each of the 90 radial directions.

The performance of the different protocols was compared using inner, outer, and overall vessel acutance (i.e., perceived sharpness) values, which were calculated from a semi-automatic vessel delineation algorithm.^48^ This algorithm consists of four steps: first, the image was inter-polated and unwrapped along 90 radial directions to generate an image of the vessel in polar coordinates: see Figure S3. Second, the gradient of this image was calculated along the radial direction for the identification of vessel edges based on the locations where the radial signal either increases or decreases. On those unwrapped gradient images, locations were manually selected to identify gradient minima and maxima which correspond to vessel edges (Figure S4). The resulting outlines were then visually checked on the original Cartesian image for validation (Figure 1A). Finally, quantitative metrics (described below) were derived based on the delineated VW boundaries. This boundary delineation method was previously found to achieve excellent inter- and intra-observer agreement, as well as high reproducibility of results on repeated measurements of the same vessel from different scan sessions.^48^ The latter makes this a particularly useful approach for the quantitative comparison of data acquired from the same volunteers in separate scan sessions (at 3T and 7T).

Based on the identified vessel boundaries, three quantitative parameters^49^ related to vessel sharpness and contrast were calculated along each radial direction: the inner boundary acutance, outer boundary acutance, and directional RMS signal gradient (*G*_RMS_): see Figure 1B,C. Contrary to previous implementations, no normalization was required when calculating these metrics since analysis was performed on reconstructions calculated in SNR units.

The inner and outer boundary acutance metrics were calculated as the maximum radial contrast across the respective vessel boundaries in polar coordinates. The maximum contrast was calculated for the identified boundary locations on the interpolated images (within a radial range of 0.3 mm) and scaled to CNR/mm units. A radial range of 0.3 mm around the identified boundary locations was found to provide consistent results for repeated measurements despite slight vessel delineation variations. Note that, although the calculated inner boundary acutance corresponds to the wall-to-lumen contrast, the outer boundary acutance at each location can correspond to either wall-to-CSF or wall-to-tissue contrasts.

The third metric, the RMS signal gradient *G*_RMS_, calculates the signal change along the radial direction between the inner and outer vessel boundaries.^49–51^ It was previously found to correlate strongly with the subjective perception of sharpness.^50^ For each of the 90 radial directions, *G*_RMS_ is calculated from the radial gradient *G_i_* = SNR_*i*_ − SNR_*i*−1_ at each of the *n_i_* radial voxels across the VW:

(2) $$G_{\text{RMS}}=\sqrt{\frac{\Sigma_{i=1}^{n_{i}}G_{i}^{2}}{n_{i}}}.$$

The *G*_RMS_ is used for most quantitative comparisons in this work as it can be used as a single metric to describe vessel visibility (without separating the inner and outer boundary acutance), with the inner and outer boundary acutance reported in addition where relevant. However, all comparisons were calculated for all three metrics to confirm consistency of the results, and the resulting trends were found to be highly consistent.

The vessel acutance quantification using the tool and metrics described above were implemented in MATLAB based on original code by Biasiolli et al.^49^ The resulting code is available online (git.fmrib.ox.ac.uk/ndcn0873/acutance_TdB).

The significance of differences between protocols was assessed using paired-sample one-tailed t-tests which test the null hypothesis that the optimized version of a protocol does not result in increased vessel visibility (*G*_RMS_ or inner/outer boundary acutance). The null hypothesis is rejected and results are considered significant for *p* < 0.05.

## 3 Results

### 3.1 Simulation-based optimization

**Figure 2:**
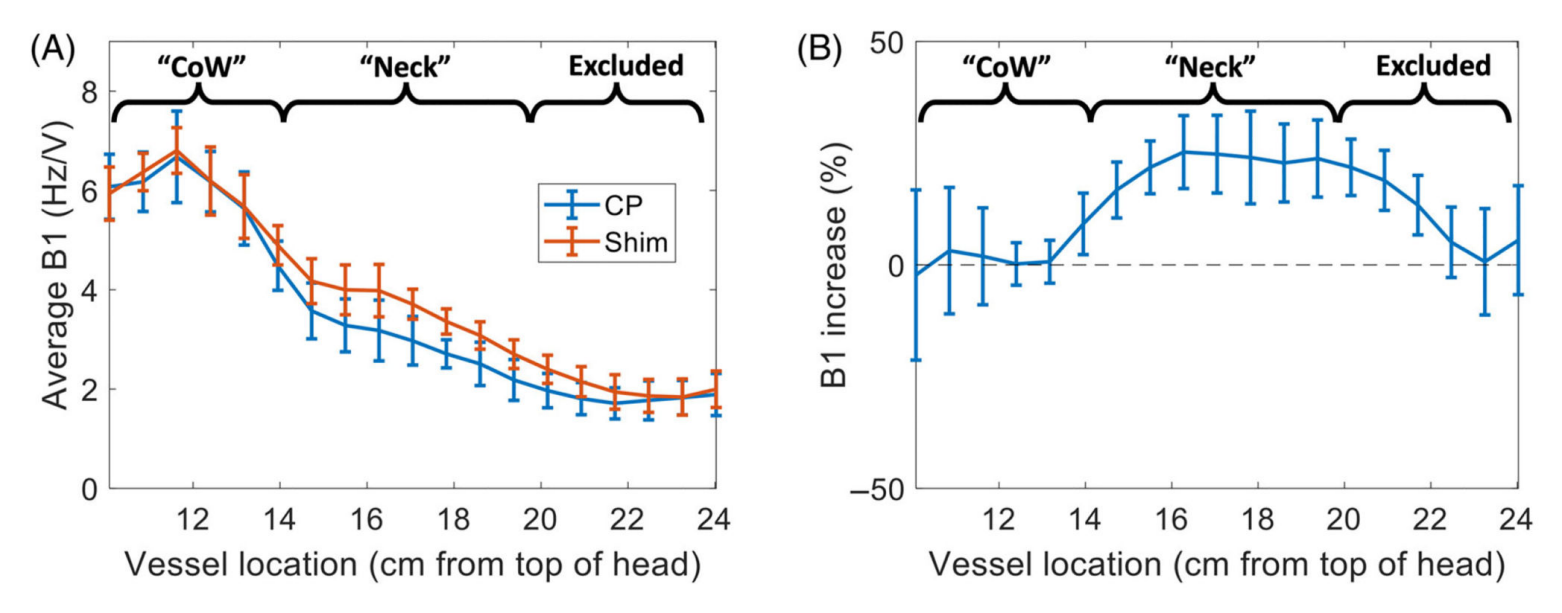
The B1^+^ magnitude at 7T in the main feeding arteries in the neck (Neck) and in the Circle of Willis (CoW) at 7T for CP-mode (blue) and the proposed neck-and-CoW RF shim (orange). Subplot (A) shows the average values and standard deviations in each slice for the 10 subjects in the multi-channel B1^+^ database; (B) shows the increase in B1^+^ magnitude in the neck region when using the RF shim instead of CP-mode.

At 7T, two different simulation-based optimizations were performed. First for implementations using CP-mode (circular polarization; as a surrogate for single-channel transmission) during both DANTE and SPACE, and second for implementations which assumed a pTx RF shim during the DANTE preparation. By increasing the B1^+^ in the neck during the DANTE preparation, this shim was intended to improve the suppression of blood in acquisitions in the downstream vasculature. Initial results indicated that using an RF shim that only maximized the B1^+^ in the feeding arteries in the neck substantially reduced the B1^+^ near the CoW, thereby reducing the CSF suppression during DANTE. Therefore, a universal “neck-and-CoW” RF shim was designed to simultaneously optimize the B1^+^ in the neck and in the CoW. As shown in Figure 2, this RF shim increases the B1^+^ magnitude in the neck without reducing the B1^+^ in the CoW, thereby improving the suppression of inflowing blood without penalizing CSF suppression. The most inferior parts of the ICAs were excluded from the RF shim calculation since the arterial transit time between those locations and the CoW is longer than the time between the start of the DANTE preparation and the sampling of the centre of k-space in the SPACE-readout (~0.4 s).

**Figure 3:**
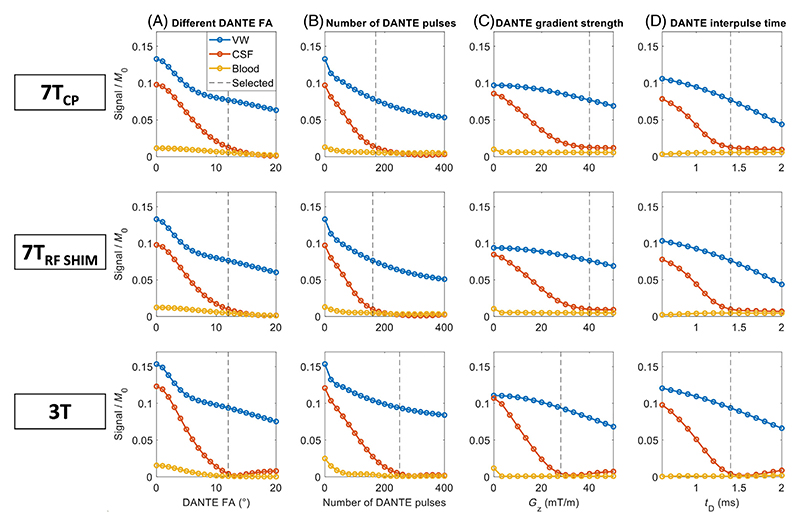
DANTE-SPACE simulation results for sweeps of the four main DANTE-parameters, simulated assuming slowly pulsating vessel walls. The simulation results are shown for 7T in CP-mode (first row), 7T using the neck-and-CoW RF shim (second row), and for 3T (third row). In each row, the subplots show the results when varying (A) DANTE flip angle, (B) number of DANTE pulses, (C) DANTE dephasing gradient strength, and (D) DANTE interpulse time. Dashed lines indicate the selected parameters for the in vivo acquisition protocol optimized for CP-mode.

The resulting single-parameter simulation sweeps at 7T without pTx, at 7T with pTx, and at 3T are shown for four DANTE parameters in Figure 3. Results are shown as the signal levels of slowly pulsating VW, CSF, and blood in turn, using the identified optimal values (indicated by dashed lines) for all other parameters. For completeness, the corresponding simulation results for fully stationary VWs can be found in Figure S5.

Simulations were also performed for the TR (optimizing for SNR/√TR) and the SPACE echo spacing. This suggested thusing a shorter overall TR at 3T than at 7T (2.10 s instead of 2.62 s), thereby also making it possible to acquire data at 3T using a lower GRAPPA acceleration factor (*R* = 3 vs. *R* = 4) without requiring a large change in the SPACE echo train length. The hardware-restricted minimum SPACE echo spacing was optimal in all cases, consistent with previous literature.^8^

An approach in which DANTE gradient directions were alternated between different parts of the acquisitions was also considered. However, simulations showed that this did not provide improved CSF suppression, as can be seen in Figure S6.

### 3.2 In vivo acquisitions

All three optimized protocols based on the simulations in Figure 3 are shown in Table 2, as well as the literature protocol used by Viessmann et al.^7^ at 7T. Data were acquired in six healthy volunteers using all four protocols.

**Figure 4:**
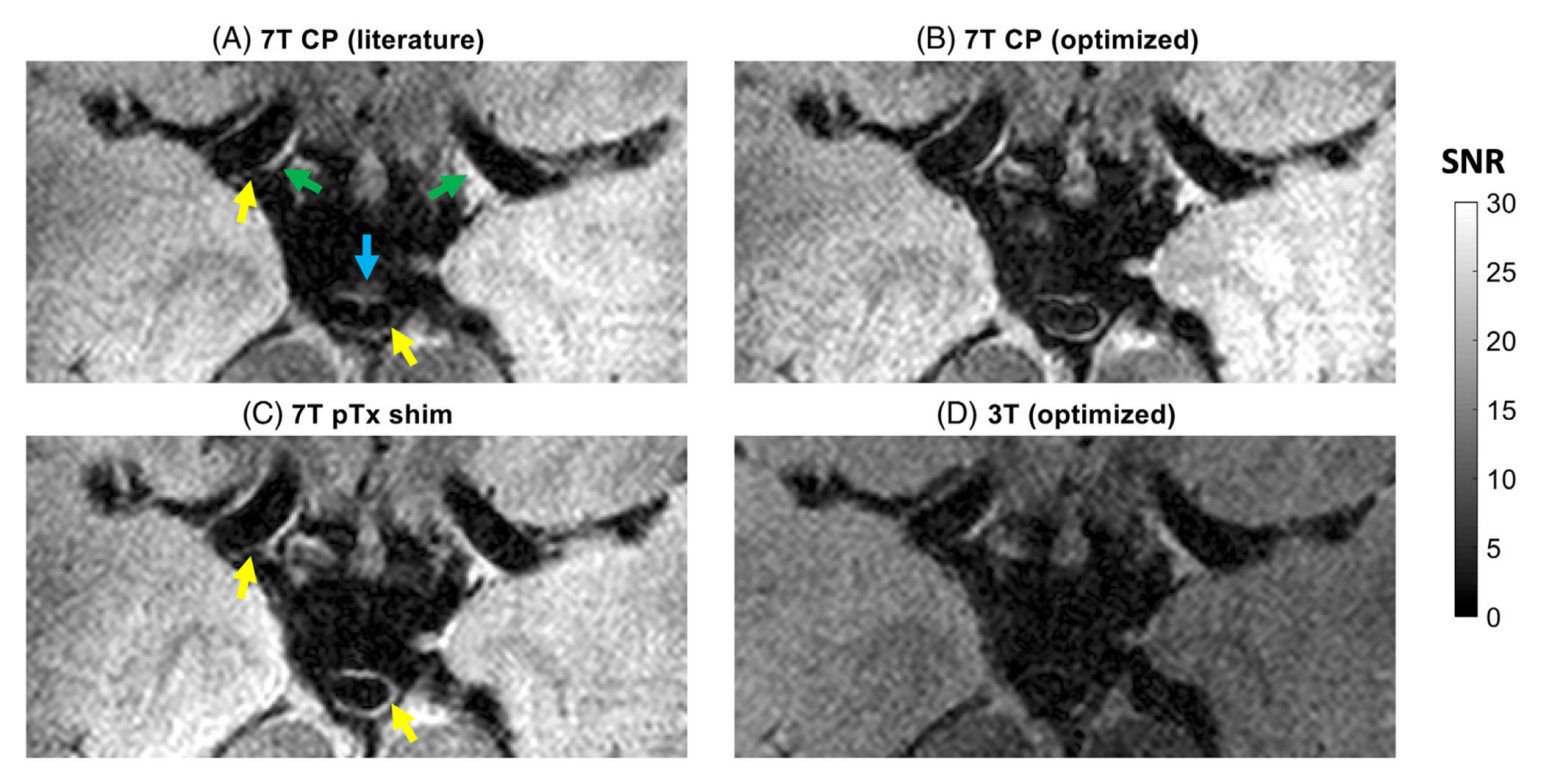
Transverse slice segment from a representative subject (26 y old male) showing the bilateral middle cerebral artery M1 segments (green arrows in [A]), and the basilar artery (blue arrow) acquired using each of the four protocols in Table 2. Yellow arrows indicate example locations with VW pulsation-induced signal attenuation in (A), for which some signal is recovered using the optimized protocol in (C).

Representative examples of data acquired using all four protocols are shown in Figure 4. The MCAs are visible in all four acquisitions (3T and 7T), while the BA is only visible at 7T. When using the optimized protocols at 7T, an increase in SNR is visible in some VW segments with lower signal when using the literature parameters, such as in the BA. All acquisitions generally achieve good CSF and blood suppression, although some heterogeneity remains. When comparing the optimizations at 7T with and without the neck-and-CoW RF shim, a slight additional reduction in blood signal can be observed in the BA and the right MCA when using the RF shim. Additional examples of acquisitions in all subjects using both the literature protocol and the optimized protocol at 7T are shown in Figure S7.

### 3.3 Quantitative comparison

**Figure 5:**
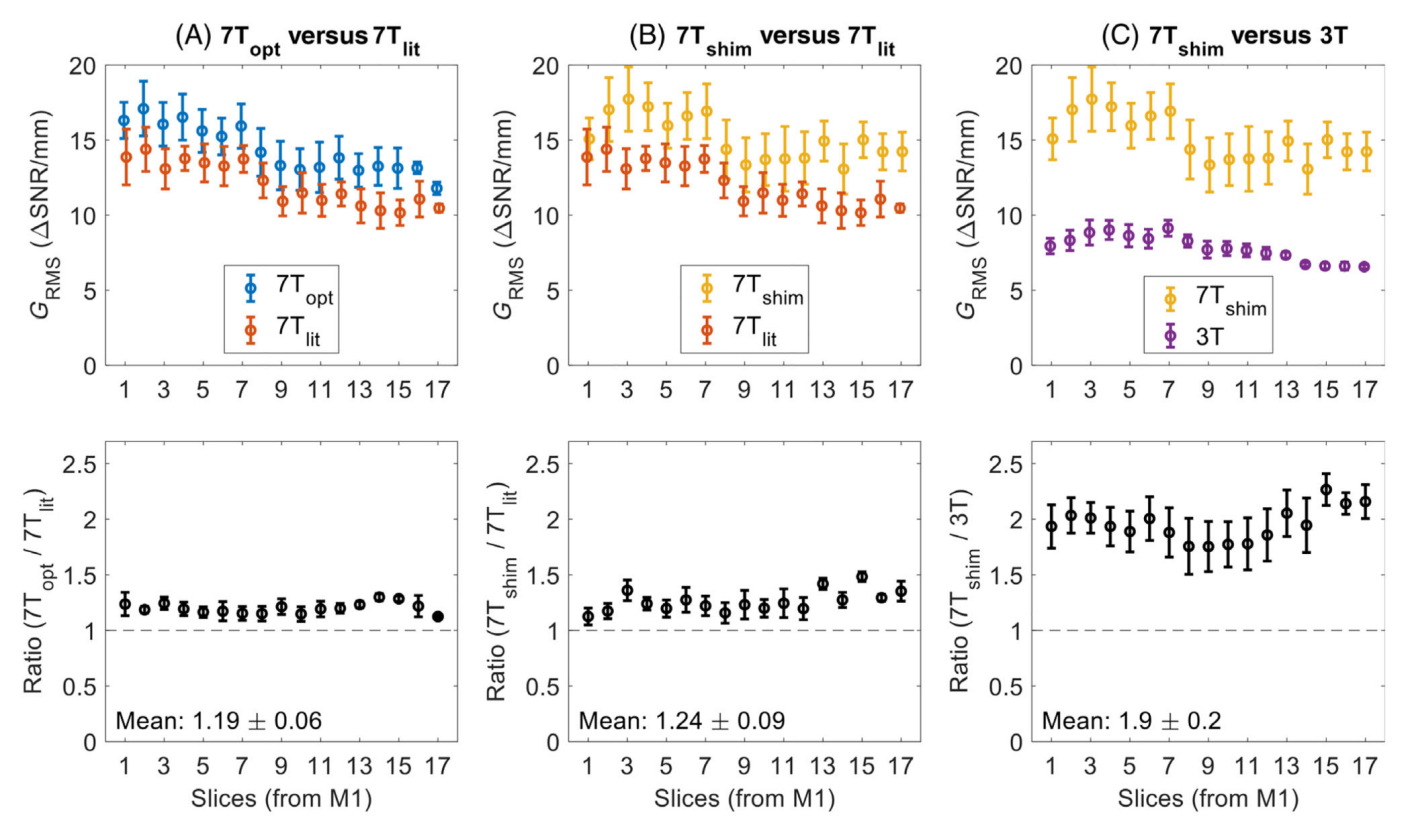
The slice-wise *G*_RMS_ of the three optimized DANTE-SPACE protocols. The matched results are shown for comparisons between acquisition using (A) CP-mode optimized (“7T_opt_”) and CP-mode literature-based (“7T_lit_”) 7T protocols, (B) RF shim-optimized (“7T_shim_”) and CP-mode literature-based (“7T_lit_”) 7T protocols, and (C) 7T RF shim-optimized (“7T_shim_”) and 3T optimized (“3T”) protocols. Reported values are the mean values and standard errors in the MCA, BA, and distal ICA of the six healthy volunteers in the 17 slices in the region shaded in yellow in Figure S8. The bottom row shows the slice-wise ratios between each pair of compared protocols.

All visible segments of the MCAs, BA, and distal ICAs in the four datasets from the six subjects were analyzed using the method described in Section 2.4. Figure 5 shows the resulting average *G*_RMS_ values at different slice locations below the MCA M1-segment in slice segments where the vessel walls are surrounded by CSF, as indicated by Figure S8. When using the CP-mode-optimized DANTE parameters instead of literature parameters at 7T (Figure 5A), the slice-wise average *G*_RMS_ increases by 19±16%, with increased values at all slice locations.

When employing the proposed universal neck-and-CoW RF shim during DANTE, Figure 5B shows a slightly higher average *G*_RMS_ increase of 24±23% relative to the literature parameter acquisitions. Again, an improvement is observed at all slice locations, although a larger relative increase is observed in lower slices. This difference is mainly driven by the inclusion of the BA, which (as is also visible in Figure 4) had a higher average *G*_RMS_ improvement than the other arteries: an increase of 36±24% when using the neck-and-CoW RF shim and an increase of 27±15% when using CP-mode optimized DANTE preparation.

Finally, as shown in Figure 5C, with an increase of 90±20%, the *G*_RMS_ is again almost two times higher in optimized acquisitions at 7T than at 3T, with consistently increased values at all slice locations.

**Figure 6:**
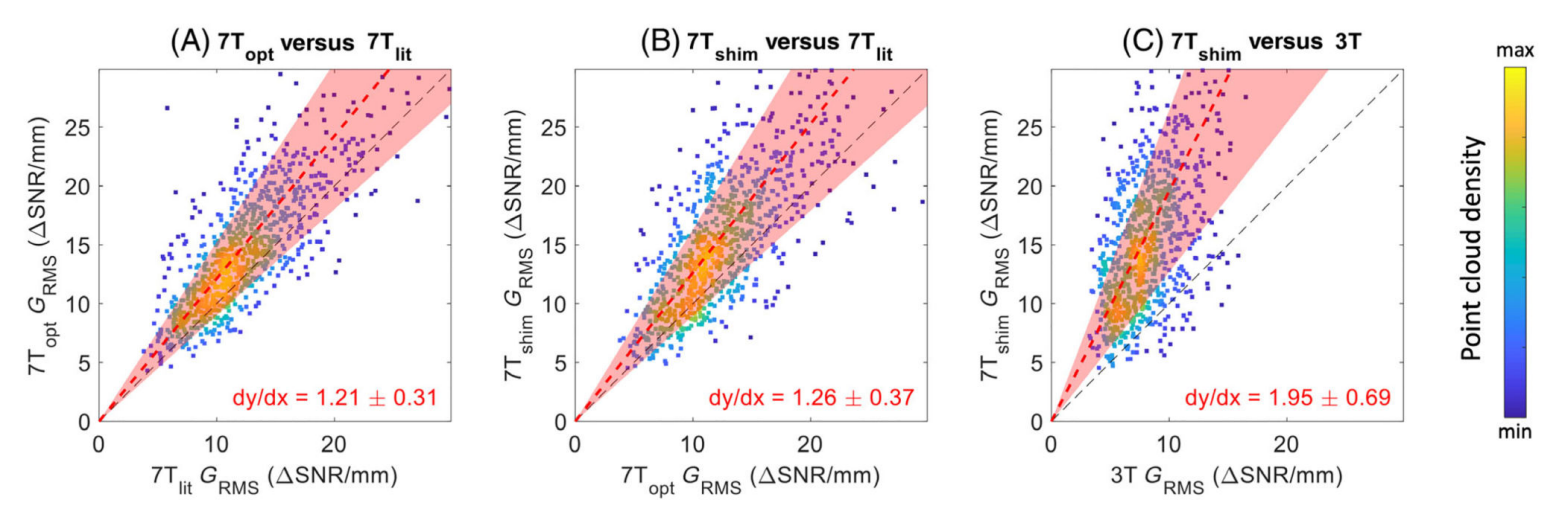
Segment-wise *G*_RMS_ correlation results for the three optimized DANTE-SPACE protocols. The results of each vessel wall segment are shown based on the average values of six radial sub-segments of 60°. Dashed red lines show the mean ratio (and 95% variance) between the respective acquisitions, with the corresponding values indicated in red. Datapoints with different colors indicate areas with different point cloud densities, resulting in overlapping datapoints.

While Figure 5 compares the slice-wise average *G*_RMS_ results, Figure 6 shows the correlation of the achieved *G*_RMS_ in the different acquisitions for each individual 60° radial sub-segment. As for Figure 5, an increased average *G*_RMS_ is observed when using the CP-mode optimized protocol (*dy/dx*>1), with a slightly larger increase still when including the neck-and-CoW RF shim during DANTE. Again, a nearly two times higher *G*_RMS_ is consistently observed at 7T versus 3T (*dy/dx* = 1.95).

**Figure 7:**
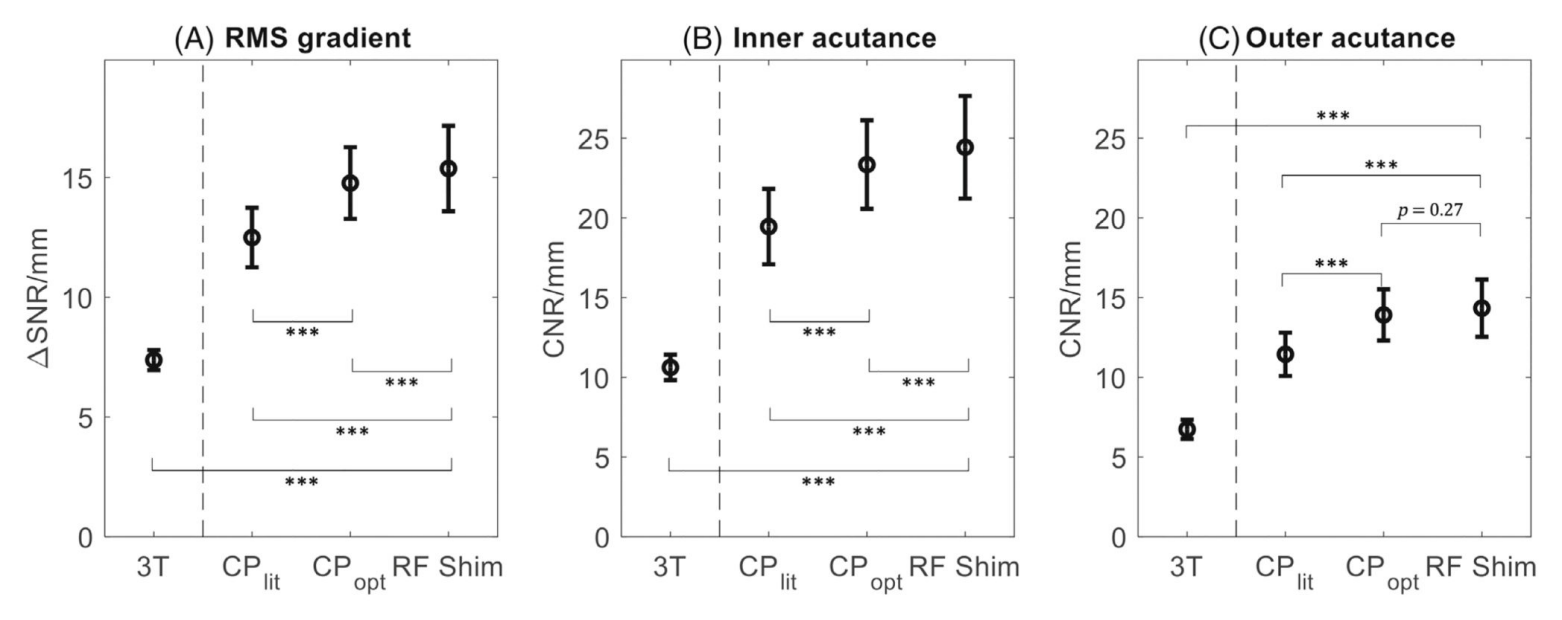
Summary results of the achieved contrasts for the four acquired protocols, compared using (A) *G*_RMS_, (B) inner boundary acutance, and (C) outer boundary acutance. Significance levels are reported for all comparisons, where three asterisks (***) denote *p* < 0.001.

The overall average *G*_RMS_ results are shown in Figure 7, along with the resulting inner and outer boundary acutance. Using all three metrics, both optimized protocols at 7T (CP-mode and using the RF shim) perform significantly (*p* < 0.001) better than the CP-mode literature protocol. The optimized protocol using the universal neck-and-CoW RF shim during DANTE achieves significantly improved *G*_RMS_ and inner boundary acutance compared to the CP-mode-optimized protocol, but no further significant increase in the outer boundary acutance is observed.

At 7T, the average improvement when using the optimized protocol with the RF shim versus literature parameters is +24±23% for the *G*_RMS_, +28±27% for the inner acutance, and + 28±33% for the outer acutance.

The average *G*_RMS_ at 7T is again nearly two times higher than at 3T, with an average difference in *G*_RMS_ of 8.0±1.7 ΔSNR/mm (*p* < 0.001). The inner and outer boundary acutance are also significantly higher at 7T, with an average difference in inner acutance of 13.8±3.2 CNR/mm (*p* < 0.001) and an average difference in outer acutance of 7.6±1.8 CNR/mm (*p* < 0.001).

## 4 Discussion

In this work, optimized versions of the DANTE preparation module for T2-weighted DANTE-SPACE VW imaging are proposed at 7T without pTx, at 7T with pTx, and at 3T. The protocols are based on simulations which compare the achieved contrasts when using different DANTE flip angles, numbers of pulses, gradient strengths, and interpulse durations.

### 4.1 Optimization at 7T

For CP-mode at 7T, Figure 3 shows that the DANTE parameters used in previous literature leave substantial room for contrast improvement for simulations assuming slowly pulsating VWs. Therefore, optimized CP-mode DANTE parameters (with 170 pulses of 12°) are proposed to improve the contrasts for pulsating VWs without substantially reducing the contrasts for stationary VW tissue relative to the literature protocol. It should be noted that the CP-mode parameters proposed here differ from the parameters used in a previous conference abstract (consisting of 200 DANTE pulses of 9°).^52^ That protocol was based on an earlier version of the simulation framework which did not include pulsatile CSF velocity variation. Since the addition of this parameter in simulations introduces periods of very low absolute velocities (near the velocity zero-crossing), it requires an increased DANTE flip angle to properly suppress CSF during all stages of the pulsatile cycle. Other studies using DANTE simulations generally assumed VW tissue to be static^4,7,12,16,20,23,24,52^ and intracranial CSF to be either static^16,20^ or constantly fast-flowing (≥2 cm/s).^7,23,24,52^ The results presented here indicate that the addition of higher-order pulsatile motion for both VW and CSF in simulations provides a more accurate representation of in vivo contrast mechanisms, and results in a substantial change in simulation-based optimal sequence parameters.

Furthermore, the results in Figure 3 show that for the four studied DANTE parameters, a strategy that alters the parameters to retain more VW signal (relative to the signal levels that are achieved using the proposed parameters) would result in the unwanted corollary of an even larger increase in CSF signal. This trade-off between suppressing CSF signal while retaining VW signal results in the maximized contrast for the DANTE parameters proposed here.

An optimized 7T protocol using a phase-only universal “neck-and-CoW” pTx shim during DANTE was also proposed. This shim increases the B1^+^ magnitude in the neck without reducing the B1^+^ in the CoW. The resulting simulations suggest that, as a result, blood suppression can be improved by 32% without penalizing the resulting CSF suppression in the region of the CoW.

Overall, the simulation results for 7T suggest that using the optimized protocols as shown in Table 2 will lead to a substantial (up to 78%) increase in the DANTE-SPACE contrasts for pulsating VWs, without reducing the contrasts for stationary VW. This also results in an 18% (CP-mode) or 23% (neck-and-CoW RF Shim) reduction in the SAR contribution of the DANTE preparation. For the full sequence, this corresponds to a SAR reduction of 6% or 8%, respectively.

The acquired slice segments in Figure 4 indicate experimental signal changes that are consistent with the simulation-based predictions: higher VW signal levels are visible when using the optimized protocols (in particular in parts of the vessels with lower initial signal levels), and a further reduction in blood signal is visible when using the neck-and-CoW RF shim. Figures 5A,B and 6A,B confirm that those contrast improvements are consistently detected for vessels in all slices and in all parts of the vessels. A substantial variation in both the achieved contrasts and the changes in contrasts is visible (Figure 6A,B) for both optimized protocols at 7T. This could be explained by a combination of variation in pulsatile behavior (velocity and direction) of VW and CSF in different anatomical areas, and by the outer VW contrasts being dependent on anatomical locations (e.g., well-defined vessel-to-CSF interfaces or poorly defined vessel-to-tissue interfaces).

Despite this variation, Figure 7 confirms that both optimized protocols result in significantly improved contrasts (*G*_RMS_, inner acutance, and outer acutance) relative to the literature protocol. When employing the neck-and-CoW RF shim versus CP-mode during DANTE, a small but significant further incremental improvement is achieved for the *G*_RMS_ and inner acutance, but not for the outer acutance. This is consistent with the hypothesis that the RF shim improves the blood suppression and therefore increases the inner VW acutance, as well as the *G*_RMS_ (which is sensitive to both the inner and outer VW acutance).

### 4.2 Optimization at 3T

The DANTE-SPACE sequence parameters at 3T were first optimized using simulations, after which the resulting achieved vessel delineation was compared between the optimized protocols at 3T and 7T.

Figure 3 shows the simulation results for various DANTE parameters, which directly affect the degree of CSF signal suppression. Compared to the result of the corresponding optimizations at 7T (also shown in Figure 3), the biggest difference in the resulting optimized parameters is an increased number of DANTE pulses at 3T (250 pulses at 3T vs. 170 pulses at 7T). This is mainly due to the lower maximum gradient strength available on the scanner used at 3T and the longer T2 relaxation time of CSF, both of which result in a slower suppression of the CSF signal. In addition to those differences in DANTE-SPACE parameters, acquisitions at 3T also require the addition of a spatial saturation band inferior to the acquired FOV to prevent longitudinal (head–neck) wraparound.

The registered CoW segment in Figure 4, acquired using T2-weighted DANTE-SPACE at 3T and 7T, shows that rigid-body registration limited to 3D shift and in-plane rotation achieves sufficient registration. Good qualitative agreement was observed for all volunteers after registration using those 4 out of 6 possible degrees of freedom. This is consistent with the expectation of there being only small absolute displacements arising from any remaining rotations near the CoW due to its anatomical location near the centre of rotation.

Consistent with the simulations in Figure 3 and 4 shows that the DANTE-SPACE acquisitions at 3T achieve good CSF suppression, while a substantial SNR reduction is visible throughout the soft tissue, and in particular in the VWs. When comparing the DANTE-SPACE acquisitions at 3T and 7T, similar CSF suppression can be observed but with substantially higher VW SNR at 7T. This allows for better delineation of all visible vessels at 7T.

Quantitatively, T2-weighted DANTE-SPACE images at 7T achieve significantly higher vessel contrasts at all slice locations (Figure 5C) and for the vast majority of the vessel segments (Figure 6). Overall, a near two-fold increase in *G*_RMS_ is observed at 7T compared to 3T, with significant improvements in both the inner and outer vessel acutance.

The proposed T2-weighted 3T DANTE-SPACE protocol uses a long DANTE preparation consisting of 250 pulses with relatively high flip angles of 12°, which was required to achieve sufficient CSF suppression. For comparison, Zhang et al.^20^ previously acquired T2-weighted DANTE-SPACE data at 3T using 150 DANTE pulses of 8°. They concluded that the resulting CSF signal suppression was insufficient and obstructed VW visualization. This observation is consistent with the results of additional simulations using our simulation framework with the parameters as used by Zhang et al.,^20^ which suggest that their DANTE preparation results in a reduction in CSF signal of only 51%, as opposed to a 96% reduction when simulating using the parameters proposed here.

Compared to T2-weighted DANTE-SPACE, T1-weighted DANTE-SPACE protocols^12–19,53^ require shorter TRs and TEs, resulting in shorter scan times to achieve a similar resolution. T1-weighted acquisitions also benefit from lower native CSF signal, and the use of DANTE for further CSF suppression is more efficient when using shorter TRs due to the reduced time for signal recovery between consecutive DANTE-SPACE modules. This explains why good dark-CSF contrast can be achieved using less aggressive DANTE preparation (e.g., 150 pulses of 8°,^13,14,16^ 94 pulses of 13°,^19^ or 100 pulses of 10°^12^) for T1-weighted DANTE-SPACE protocols. Less aggressive DANTE preparation also results in minimal VW signal suppression, whereas using the greater degree of DANTE preparation that is required for T2-weighted DANTE-SPACE we observe a substantial loss of VW signal (both in simulations and in vivo). This suggests that DANTE preparation might not be the ideal CSF suppression method for T2-weighted SPACE protocols at 3T, where T2-dependent inversion-recovery approaches (such as T2IR^20,54^) have previously been found to provide CSF suppression with minimal VW signal reduction. At 7T, DANTE-SPACE benefits from more efficient CSF suppression than at 3T (requiring fewer DANTE pulses), while methods such as T2IR would be less feasible at ultra-high field due to high SAR requirements (2.4× higher than the proposed DANTE preparation). Therefore, whereas DANTE preparation is a good option for CSF-suppression in T1-weighted and T2-weighted VWI at 7T, better options might be available for T2-weighted imaging at lower field strengths.

### 4.3 Further considerations

All data used in this work were acquired from healthy volunteers without any pathology or known vessel disease. Viessmann et al.^7^ scanned one patient with MCA stenosis using T2-weighted DANTE-SPACE at 7T, demonstrating the potential of the sequence for depicting intracranial VW pathology. However, further clinical validation for our recommended parameters is still required. Based on pathological VW simulations (not shown), the contrasts between clinically relevant plaque components are expected to remain consistent when using the optimized protocols proposed here. However, due to VW pathology and aging,^55^ reduced VW motion might be expected in clinical populations. That would have the benefit of reducing VW attenuation, resulting in improved DANTE-SPACE contrasts in patient populations.

Furthermore, the protocol used here required an acquisition time of over 11 min and used anisotropic voxels, neither of which is desirable in a clinical setting. Therefore, future work should explore the potential of improving the readout properties of the T2-weighted DANTE-SPACE sequence (including the k-space sampling, SPACE echo trains and corresponding TEs), to facilitate faster acquisitions while still providing the SNR and resolution required for intracranial vessel wall imaging. Figure S9 shows indicative examples of isotropic T2-weighted DANTE-SPACE acquisitions at 7T with clinically feasible scan durations, including the potential use of CAIPIRINHA (Controlled Aliasing in Parallel Imaging Results in Higher Acceleration)^56^ sampling for further acceleration.

## 5 Conclusions

DANTE-SPACE simulations which include physiological variations, such as CSF pulsation, can be used to improve DANTE parameter optimization. At 7T, this provides improved DANTE-SPACE contrasts over a previous literature protocol. If parallel transmit capability is available, a neck-and-CoW RF shim during DANTE can be used for additional suppression of inflowing blood, resulting in a further improvement in delineation of the inner VW.

At 3T, simulations indicate that T2-weighted DANTE-SPACE vessel wall imaging requires more DANTE pulses to achieve sufficient CSF suppression. This leads to a substantial reduction in VW signal, resulting in vessel contrasts which are 2× lower than at 7T. Therefore, T2-weighted DANTE preparation might be more suitable for VWI at ultra-high field strengths or with different contrasts.

## Supplementary Material

Additional supporting information may be found in the online version of the article at the publisher’s website.

Supplementary file

Supplementary Figure

## Figures and Tables

**Table 1:** Overview of the relaxation parameters, mean absolute velocities, and diffusion coefficients used for the simulation of vessel wall tissue, CSF, and blood.

Parameter	Unit	3T		7T
T1 (VW)	ms	1227		1628
T1 (CSF)	ms	4019		4019
T1 (blood)	ms	1779		2290
T2 (VW)	ms	55		46
T2(CSF)	ms	517		311
T2 (blood)	ms	122		100
Mean velocity (VW)	cm/s		0.054	
Mean velocity (CSF)	cm/s		0.367	
Mean velocity (blood)	cm/s		24.0	
Diffusion coefficient (VW)	mm^2^/s		0	
Diffusion coefficient (CSF)	mm^2^/s		3 × 10^–3^	
Diffusion coefficient (blood)	mm^2^/s		3 × 10^–3^	

Abbreviations: VW, vessel wall.

**Table 2:** Overview of the parameters used for the four acquired DANTE-SPACE protocols.

Parameter	Unit	7T_CP_ (Literature)	7T_CP_ (Optimized)	7T RF Shim	3T
Scan time	min	11:32	11:32	11:32	11:31
TR	s	2.62	2.62	2.62	2.10
Acquired resolution	mm	0.47×0.47×1	0.47×0.47×1	0.47×0.47×1	0.47×0.47×1
Matrix size (acq.)		512×384×176	512×384×176	512×384×176	512×384×176
GRAPPA factor		4	4	4	3
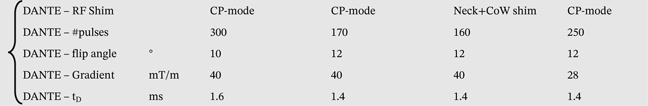					
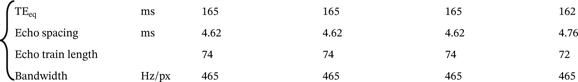					

Abbreviations: CP = circular polarization; CoW = Circle of Willis.

## Data Availability

The DANTE-SPACE simulation framework used here is openly available online at git.fmrib.ox.ac.uk/ndcn0873/dantespace_epg. The MATLAB code for vessel delineation and acutance quantification is available at git.fmrib.ox.ac.uk/ndcn0873/acutance_TdB, together with (simulated and in vivo) example data.
