# Supplementary material for: Simulation-based optimization and experimental comparison of intracranial T2-weighted DANTE-SPACE vessel wall imaging at 3T and 7T
Source: Magn Reson Med. Author manuscript; Available in PMC 2026 Feb 23. (PMC7618771; doi:10.1002/mrm.30203)
Supplement: Supplementary file [file EMS212591-supplement-Supplementary_file.docx]

**Supporting Information for***“Optimization and comparison of intracranial T2-weighted DANTE-SPACE vessel wall imaging at 3T and 7T”*

Matthijs de Buck, Aaron Hess, and Peter Jezzard


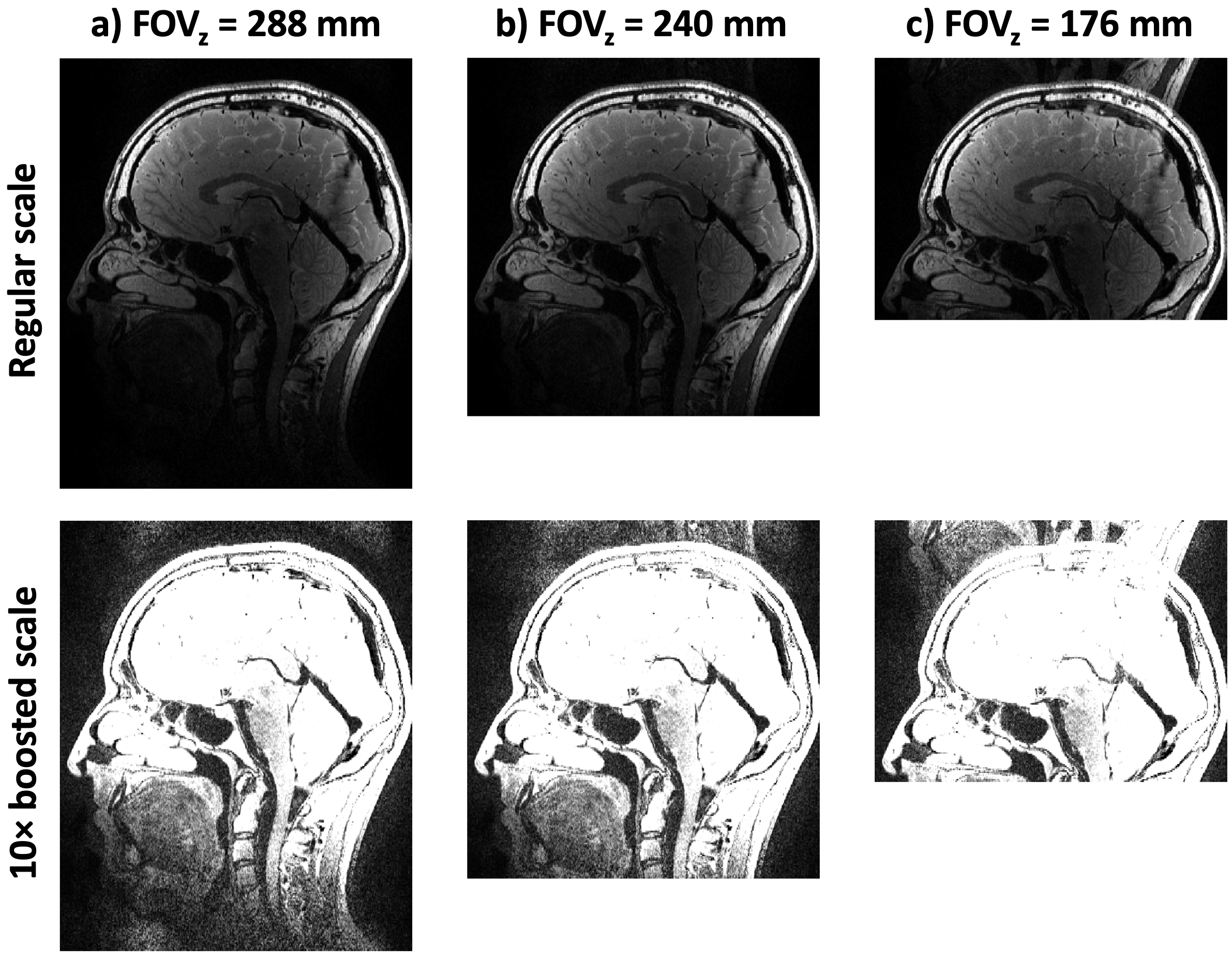


**Supporting Information Figure S1:** Central sagittal DANTE-SPACE slices acquired at 3T using a longitudinal field-of-view of **(a)** 288 mm, **(b)** 240 mm, or **(c)** 176 mm. The top row shows reconstructions using a regular intensity scale (in arbitrary units), and the bottom row shows the same data on a 10× boosted scale to emphasize the increased bottom-to-top wraparound for smaller FOVs in absence of a regional saturation band.


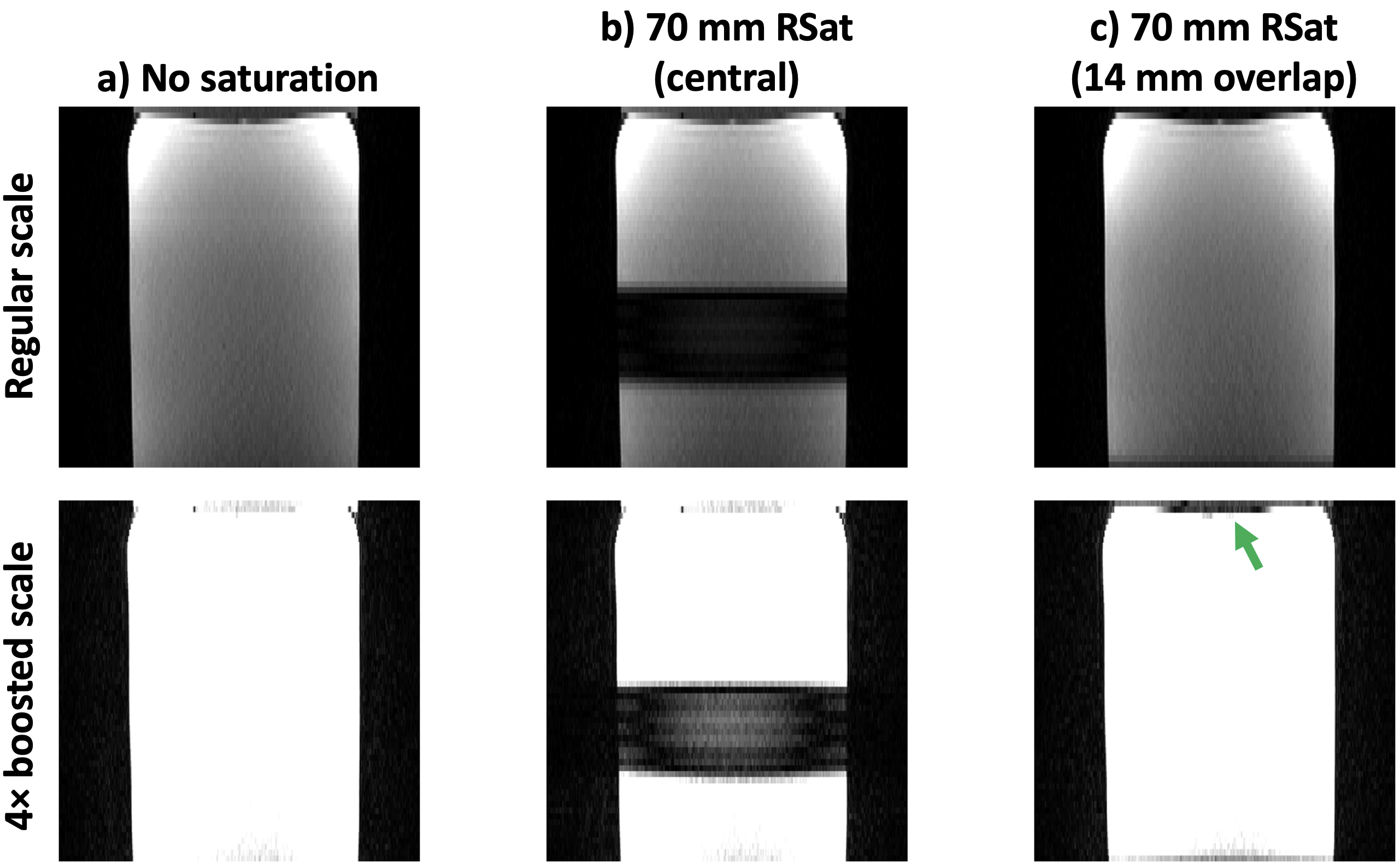


**Supporting Information Figure S2:** Central sagittal DANTE-SPACE slices acquired at 3T in a phantom for acquisitions using **(a)** no spatial saturation, **(b)** a saturation region (RSat) of 70 mm in the centre of the phantom, and **(c)** a saturation region of 70 mm located inferior to the FOV with 14 mm overlap. The top row shows reconstructions using a regular intensity scale (in arbitrary units), and the bottom row shows the same data on a 4× boosted scale. The green arrow in (c) indicates the region where longitudinal wrap-around is suppressed when using the proposed saturation band.


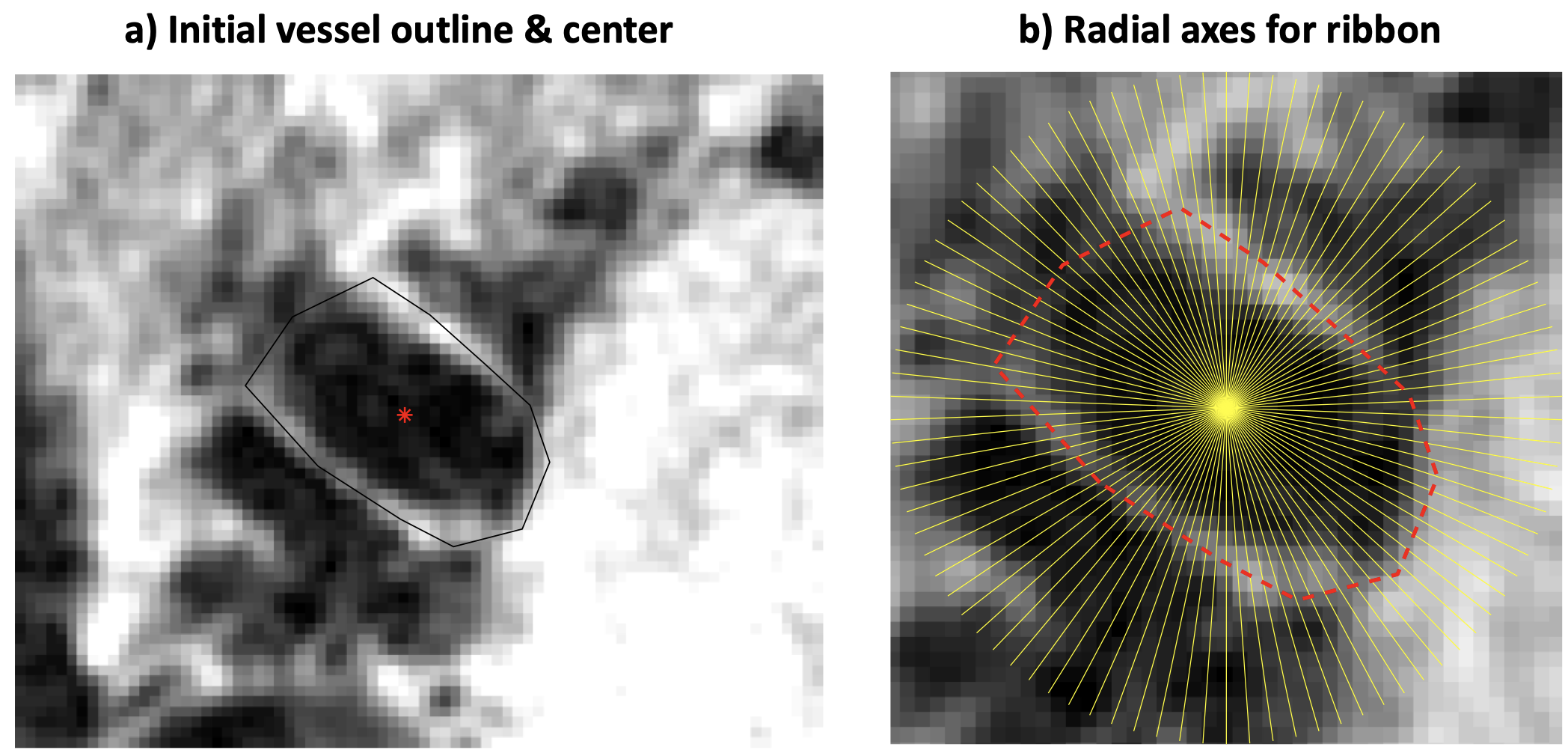


**Supporting Information Figure S3:** The first step of the vessel delineation algorithm: **(a)** A rough outline of the vessel is manually indicated (black line), from where the centre of the vessel area is estimated (red asterisk). **(b)** From this central location, 90 radial lines are extracted at 4° intervals (yellow lines) on a four-fold interpolated version of the image segment.


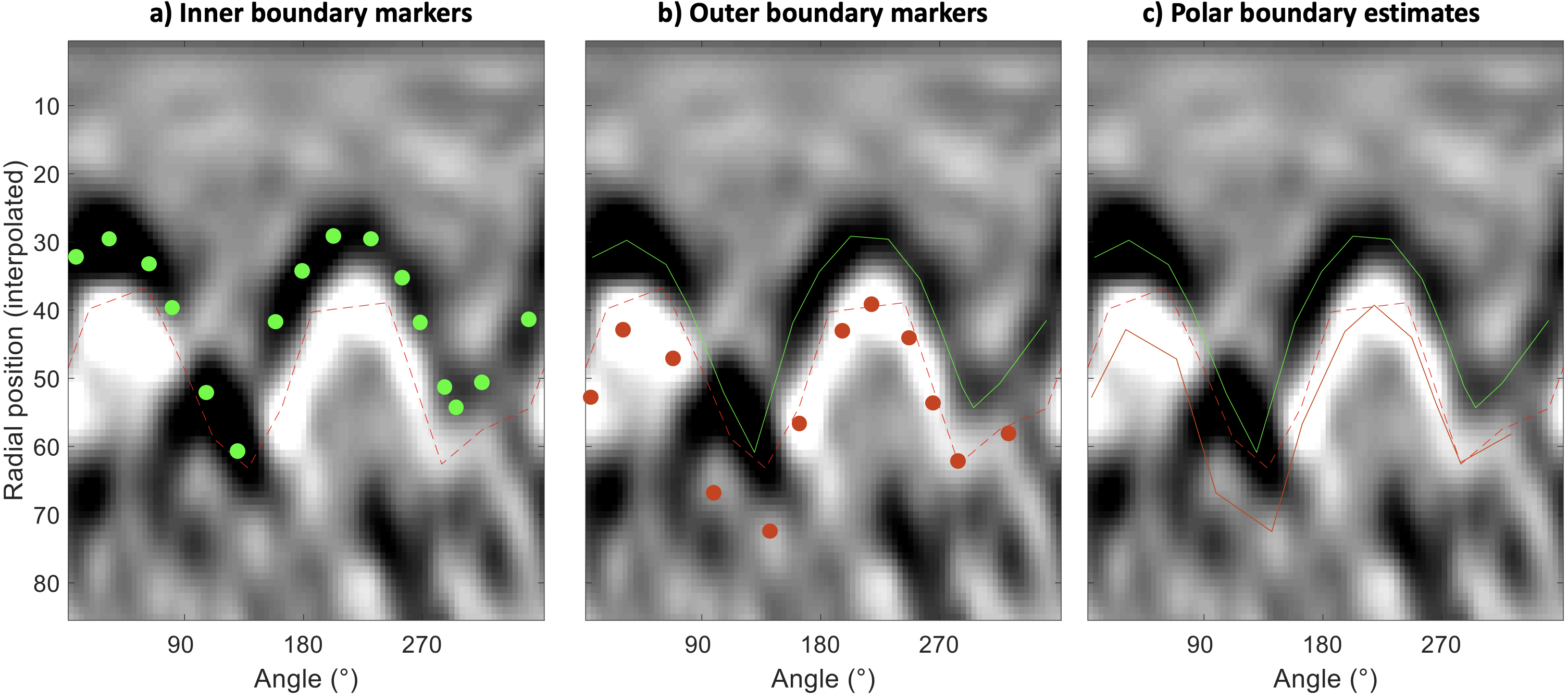


**Supporting Information Figure S4:** Vessel delineation on the polar gradient images. For each subplot, the y-axis indicates the distance from the centre of the vessel (central point corresponding to the top of the 2D images) and the y-axis indicates the clockwise angle, starting at 0° denoting vertical up (i.e., 12 o’clock) in Figure S3b. Greyscale images show the centre-out gradient values along each of the 90 identified vessel locations, with black corresponding to a signal increase and white to a signal decrease. The inner boundary is expected at the minimum points of the “troughs”. The outer boundary is expected at the maximum points in the “peaks”. The dashed red line corresponds to the initial vessel estimate drawn in the first step and serves as a guide to the eye. **(a)** Markers are manually placed at expected inner boundary locations (green circles) and **(b)** expected outer boundary locations (orange circles). The green line in (b) indicates the inner boundary estimates from (a). **(c)** The resulting manually indicated boundary estimates (inner boundary green line; outer boundary continuous orange line).


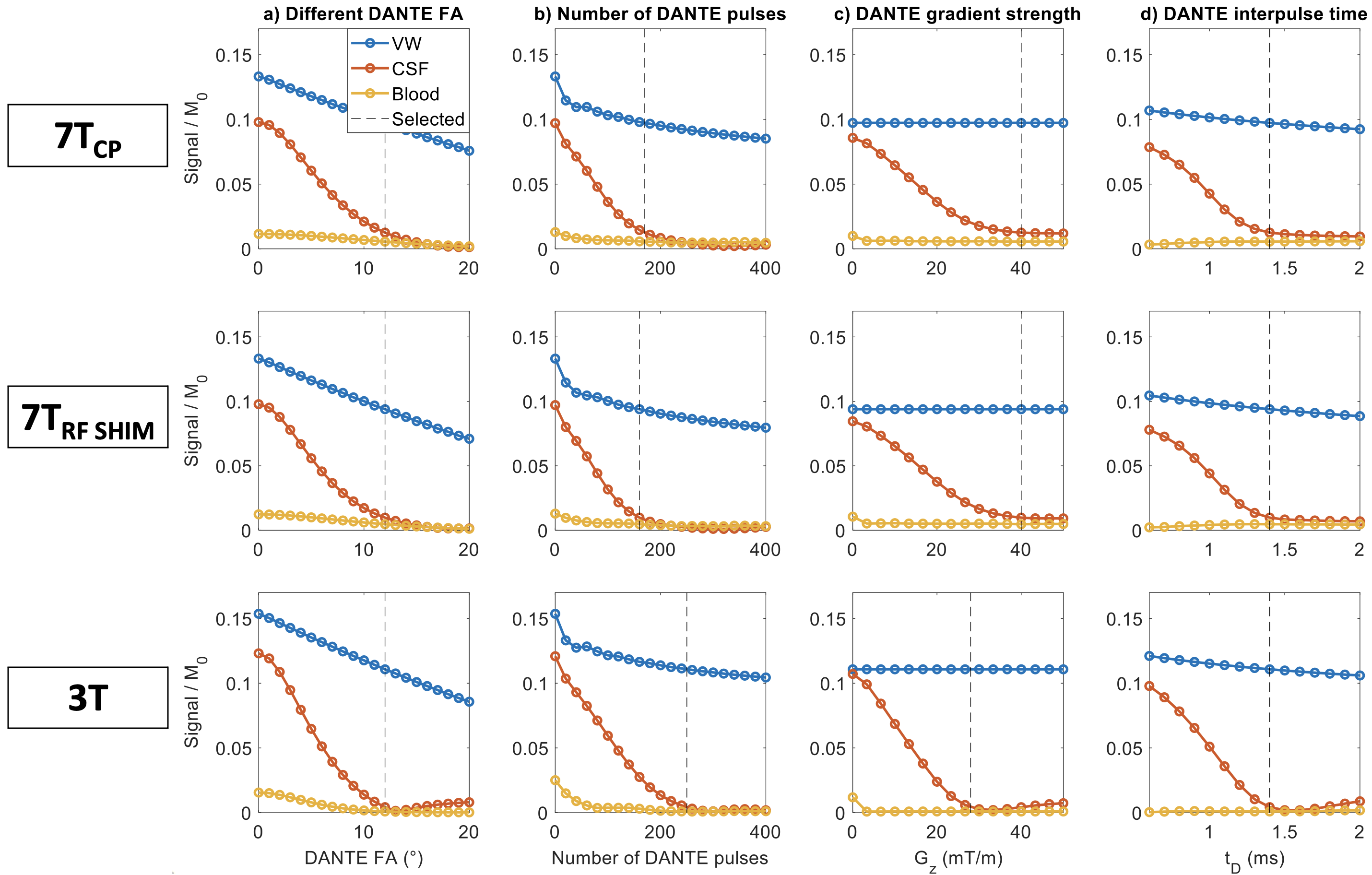


**Supporting Information Figure S5:** DANTE-SPACE simulation results for sweeps of the four main DANTE-parameters, simulated assuming stationary vessel walls (as opposed to the slowly pulsating vessel walls in Figure 4 of the main manuscript). The simulation results are shown for 7T in CP-mode (first row), 7T using the neck-and-CoW RF shim (second row), and for 3T (third row). In each row, the subplots show the results when using various **(a)** flip angles; **(b)** numbers of pulses; **(c)** dephasing gradient strengths; and **(d)** interpulse times. Dashed lines indicate the selected parameters for the in vivo acquisition protocol optimized for CP-mode.


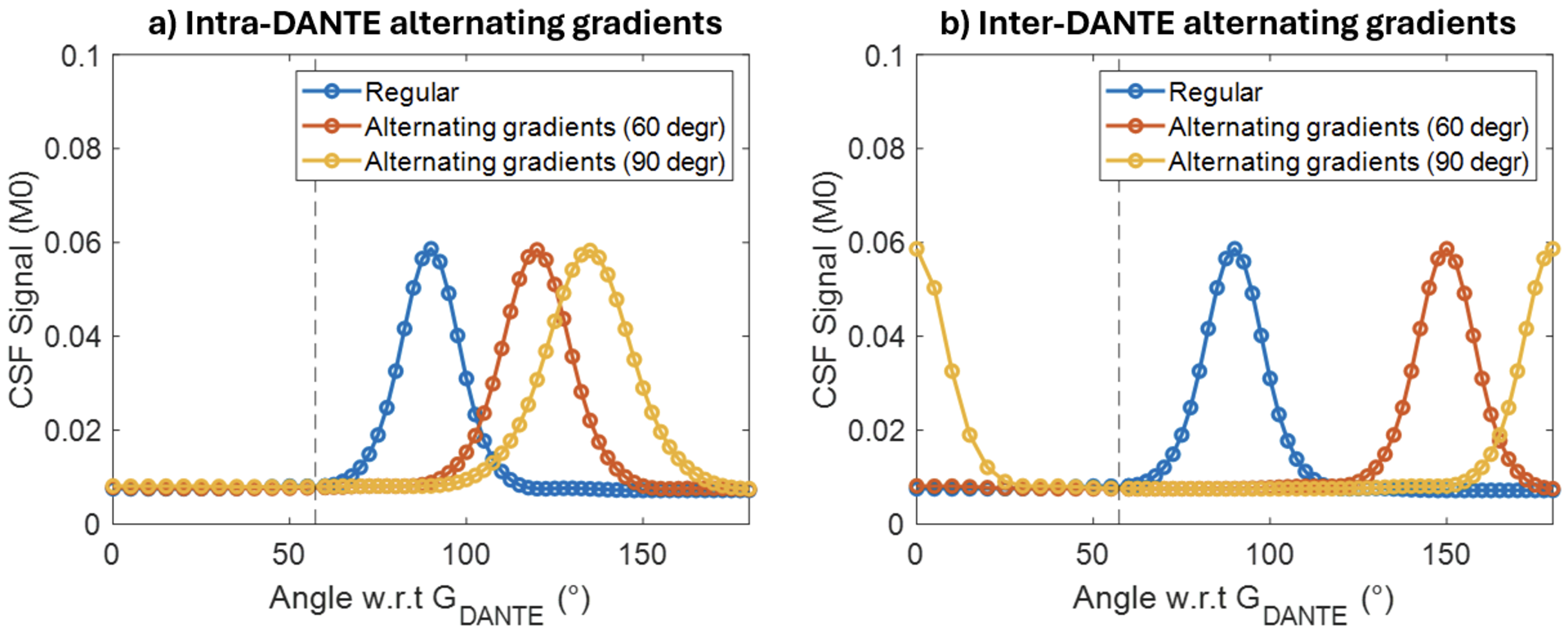


**Supporting Information Figure S6:** Simulated signal for various CSF flow directions when assuming two different methods of alternating the DANTE gradient directions: for every pair of pulses within a DANTE pulse train (a; “intra-DANTE alternating”) and for every full DANTE pulse train (b; “inter-DANTE alternating”). The blue data show the results of conventional DANTE-SPACE simulations for different angles between the DANTE gradient vector and the CSF flow direction, highlighting that flow perpendicular to the effective gradient direction will not be suppressed. The orange and yellow data show the results when alternating between DANTE gradient vectors which differ by either 60° or 90°.

When using intra-DANTE alternating gradients (a), the results indicate non-attenuated signal for flow directions in the middle of the two alternating directions. This is a result of an equal amount of dephasing and rephasing occurring at the unsuppressed directions when using intra-DANTE alternating gradients. For inter-DANTE alternating gradients (b), the alternating DANTE gradients do not have a large carry-on affect between successive TRs as the DANTE gradients only affect the transverse magnetization, but the T2 is much shorter than the full TR (of 2.62 seconds). This means that the suppressed CSF flow directions will alternate along with the alternating gradient directions in different TRs, which in addition to leaving unsuppressed flow directions could potentially result in wraparound-like signal copies of the unsuppressed CSF due to contrast variation between alternating k-space readouts.


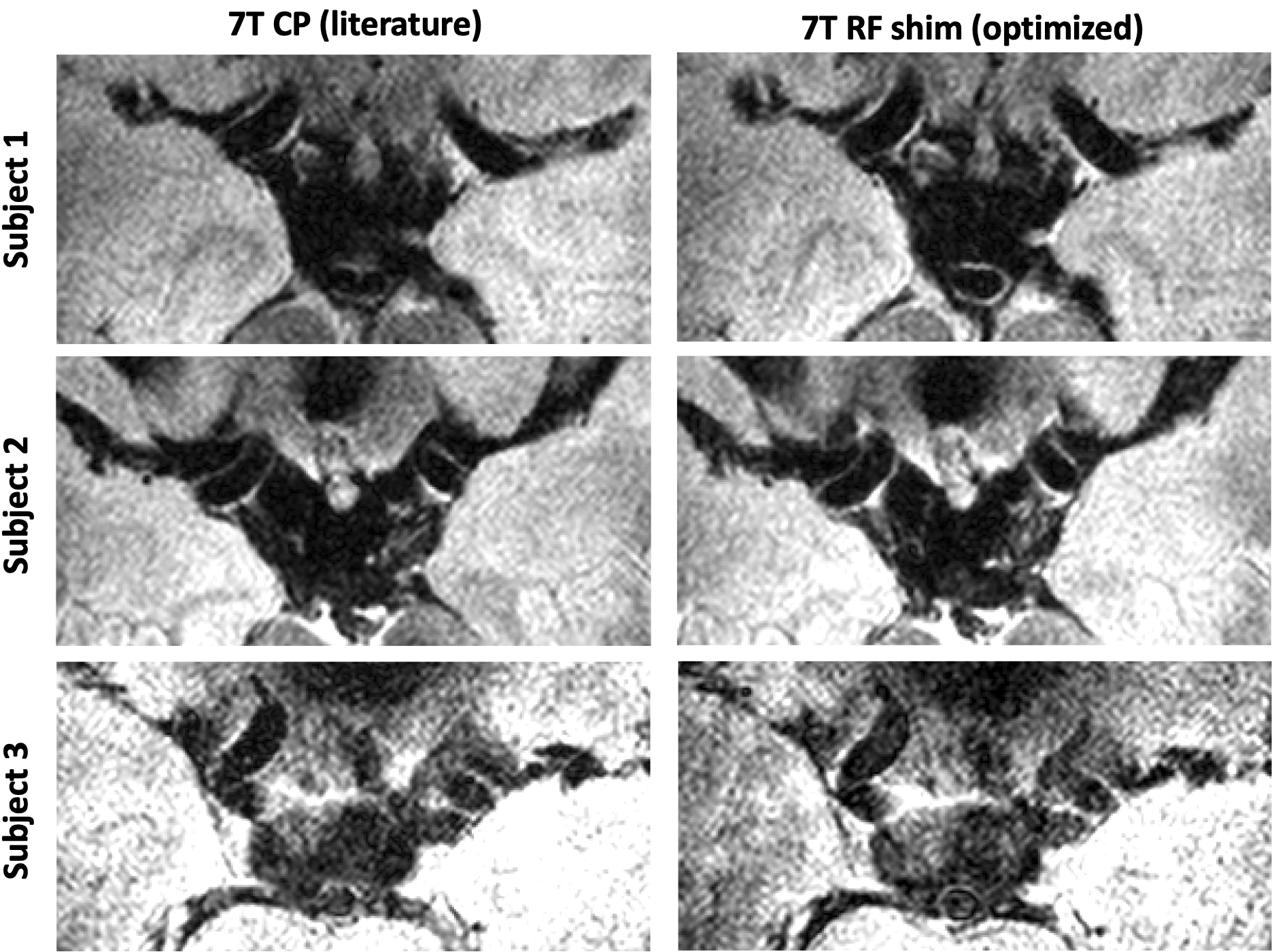


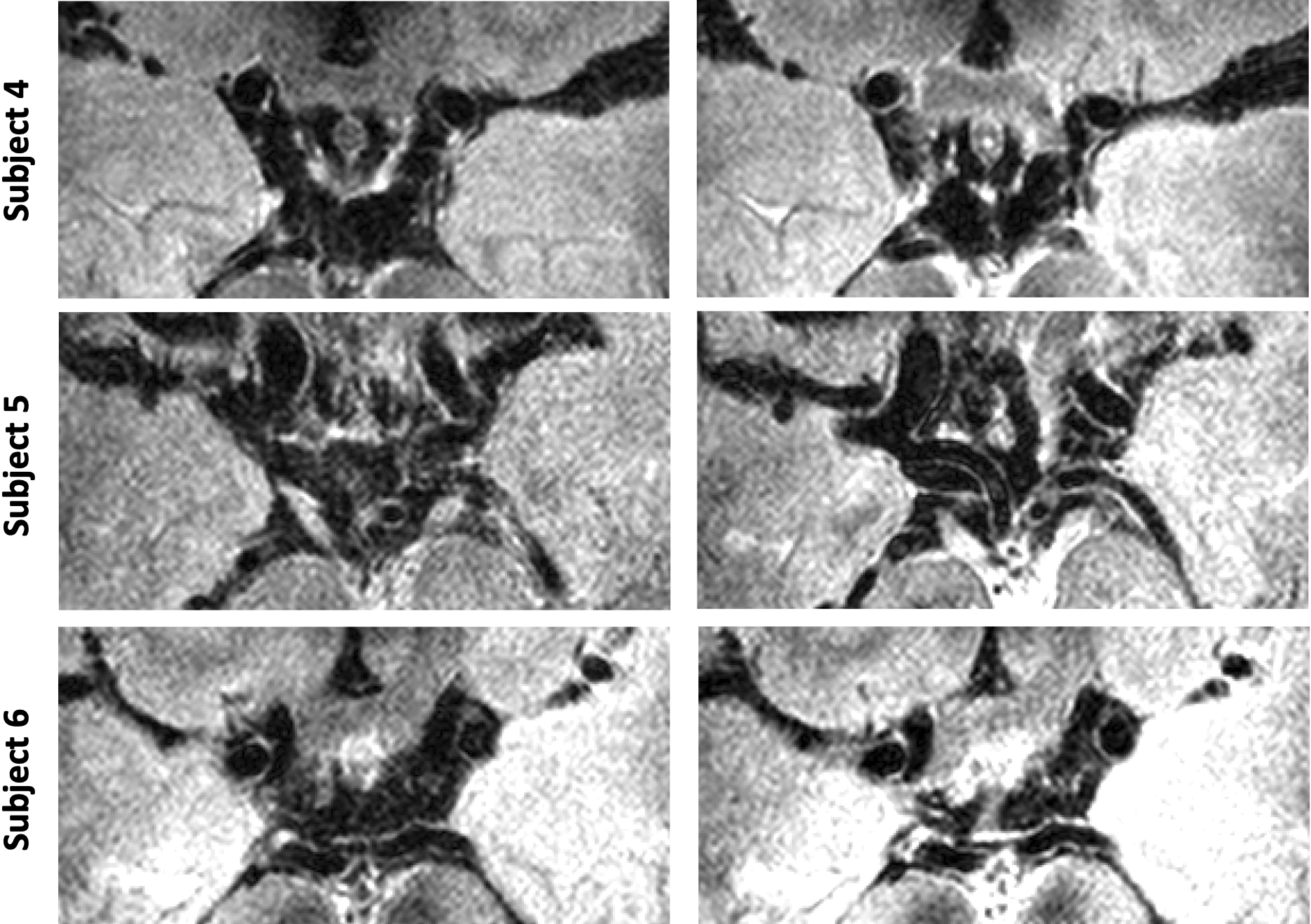


**Supporting Information Figure S7:** Example slice segments in all six healthy volunteers, using both the literature protocol at 7T (left column) and the optimized DANTE-SPACE protocol at 7T with pTx shims (right column). Note that for both acquisitions, the image quality in Subject 3 appears to be compromised by gross subject motion.


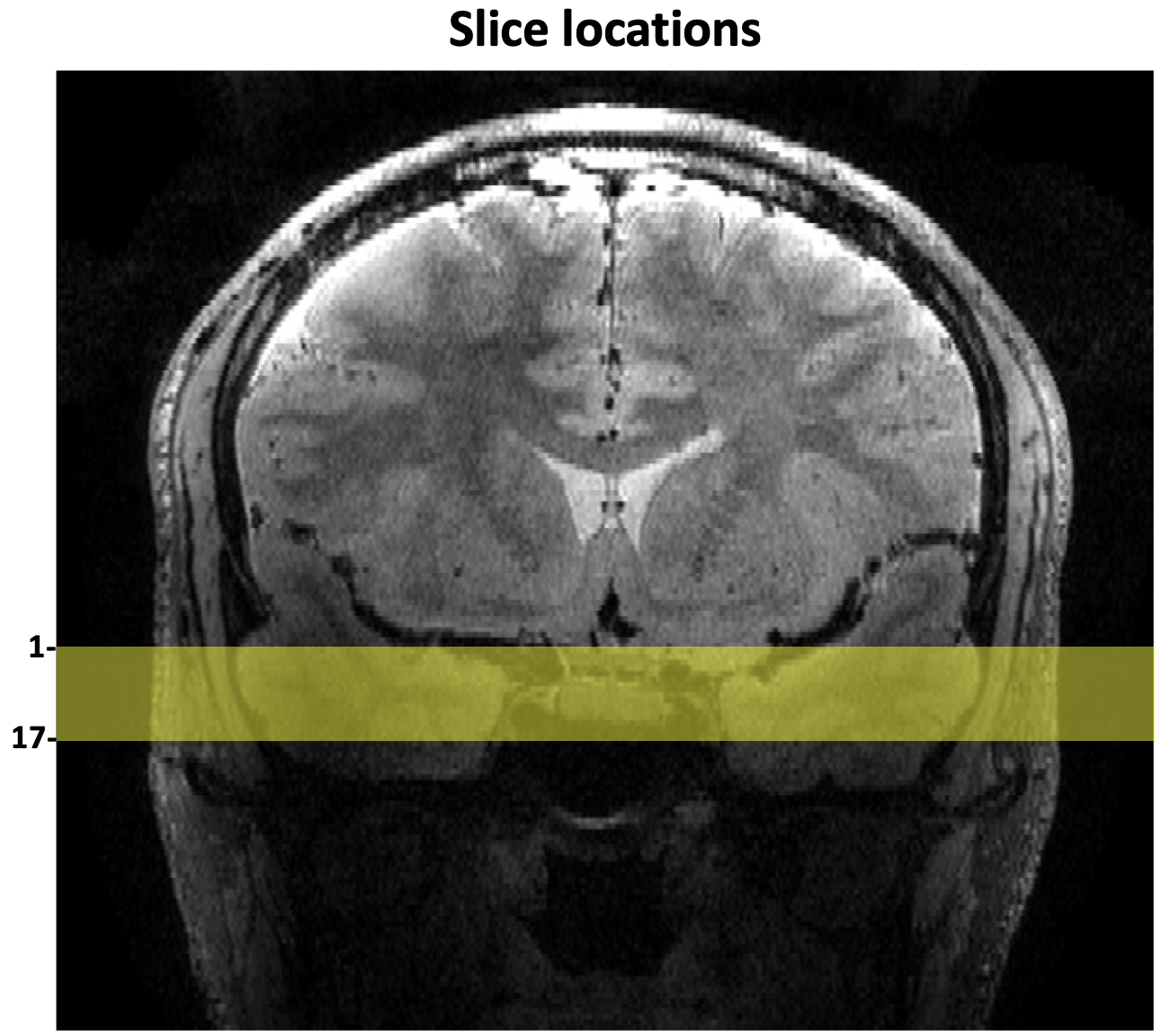


**Supporting Information Figure S8:** The 17 slices used for quantification of the slice-wise $G_{RMS}$ of the various DANTE-SPACE protocols, as shown in Figure 5 of the main manuscript. The reported values are the mean values and standard errors in the MCA, BA, and distal ICA of the 6 healthy volunteers in the 17 slices in the region shaded in yellow shown here.


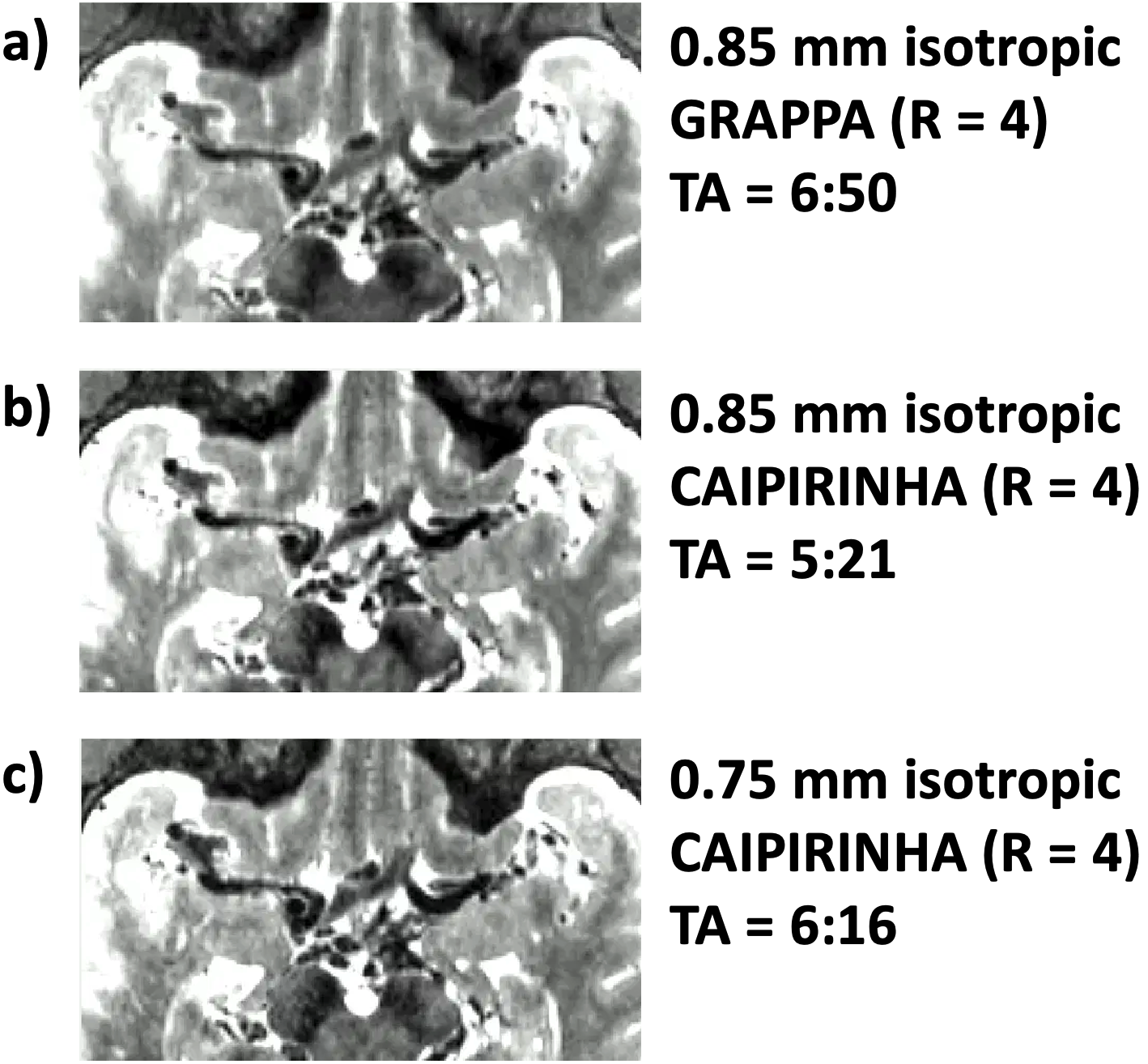


**Supporting Information Figure S9:** Example axial segments of DANTE-SPACE acquisitions with isotropic SPACE readout protocols, using more clinically desirable scan durations (compared to the protocol of 11:32 as used in the main manuscript). DANTE parameters were used as in the CP-mode optimized 7T protocol in Table 2 of the main manuscript. Data were acquired in a healthy 58-year-old male volunteer. Further readout parameters include **(a)** TR/TE = 2620/159 ms, turbo factor 96, echo spacing 3.88 ms; **(b)** TR/TE = 2620/159 ms, turbo factor 96, echo spacing 3.88 ms; and **(c)** TR/TE = 2620/160 ms, turbo factor 96, echo spacing 4.00 ms.
